# Thermal Stability and Flammability of Epoxy Composites Filled with Multi-Walled Carbon Nanotubes, Boric Acid, and Sodium Bicarbonate

**DOI:** 10.3390/polym13040638

**Published:** 2021-02-21

**Authors:** Olga B. Nazarenko, Yulia A. Amelkovich, Alexander G. Bannov, Irina S. Berdyugina, Visakh P. Maniyan

**Affiliations:** 1 Tomsk Polytechnic University, 30 Lenin Av., 634050 Tomsk, Russia; an_amo@mail.ru (O.B.N.); yulia@mail.ru (Y.A.A.); 2 Novosibirsk State Technical University, 20 K. Marx Ave., 630073 Novosibirsk, Russia; irina_berdugina@mail.ru; 3 Tomsk State University of Control Systems and Radioelectronics, 40 Lenin Av., 634050 Tomsk, Russia

**Keywords:** epoxy composites, thermal degradation, flammability, carbon nanotubes, boric acid, sodium bicarbonate

## Abstract

Epoxy composites filled with 0.5 wt% of multi-walled carbon nanotubes (MWCNTs), 10 and 15 wt% of boric acid and sodium bicarbonate separately, as well as composites filled with a combination of MWCNTs-boric acid and MWCNTs-sodium bicarbonate were prepared. The thermal behavior of the prepared samples was investigated under heating in oxidative environment using thermogravimetric analysis. The hardness was measured using the Shore D hardness test. To evaluate the flammability of the samples, the ignition temperature and time-to-ignition were determined. It was concluded that sodium bicarbonate in the studied concentrations (10 and 15 wt%) is not appropriate for use as a filler capable of improving the thermooxidative stability and reducing the flammability of epoxy polymers. The improvement in the thermal properties can be achieved by using the combination of boric acid and multi-walled carbon nanotubes as fillers. The thermooxidative destruction of the samples filled with boric acid passes more slowly and more evenly via the formation of B_2_O_3_ as a result of its decomposition.

## 1. Introduction

Epoxy resins are widely used in industry as construction materials, adhesives, coatings, and matrices for advanced composites because of their superior mechanical and electrical properties, chemical resistance, and resistance to moisture [1,2,3,4]. The main disadvantage of polymer materials, including epoxy resins, is their high flammability and fire hazard [5]. The growth of polymer consumption has caused an increase in the number of fires and the resulting property damage. Therefore, the problem of reducing the flammability of polymers is of great importance [6]. One of the methods to solve this problem is the use of flame retardants as fillers to polymers. Incorporation of different types flame retardants into epoxy resin has been investigated to improve the thermal characteristics and mechanical properties of the composite [7,8]. In general, the effectiveness of flame retardant additives is achieved when used at high loading (more than 50 wt%), which degrade the physical and mechanical properties of the polymer material. The use of nanosized additives at low loading can contribute to solving the problem of reducing the flammability of polymers [9,10]. The introduction of nanoparticles into polymers leads to a change in its molecular structure and its topological and supermolecular levels, as well as the production of a significant modification of the structure and properties of the polymer material [11,12,13]. In this case, it is expected to increase the thermal stability and reduce the flammability of the modified polymer, as well as improve the physicochemical and functional properties of the composite.

To reinforce and modify the epoxy matrices, various nanofillers of organic and inorganic nature are used [14,15,16,17]. Among the various nanofillers, multi-walled carbon nanotubes (MWCNTs) have attracted great interest recently as structural reinforcements because of their outstanding properties. MWCNTs consist of multiple layers of graphite rolled onto themselves to form a tube shape. They exhibit unique mechanical and electrical properties, which have caused them to be widely studied [18,19,20]. The use of MWCNTs as fillers makes it possible to improve the properties and performance characteristics of the polymer composites based on MWCNTs significantly. Moreover, multi-wall carbon nanotubes are considered to be highly effective flame retardants mostly at low MWCNTs content (0.5 wt% or less) [21,22,23,24,25,26,27]. However, several researchers showed that the addition of MWCNTs resulted in the decrease of the thermal stability of epoxy matrices [28,29,30]. This effect is caused by the increase of the polymer thermal conductivity as a consequence of the addition of MWCNTs and the lower crosslink density of the nanocomposites. The main obstacle to flame retardancy improvement when using MWCNTs as fillers is the difficulty of forming homogeneous nanoparticle dispersion and the poor affinity of neat MWCNTs to the epoxy matrix [31,32]. To improve the dispersion state of MWCNTs in polymer matrices and nanotubes-epoxy interfacial interaction, functionalization of carbon nanotubes has been adopted. Kuan et al. reported that the incorporation of the MWCNTs functionalized with vinyltriethoxysilane into an epoxy resin increased its thermal stability [32]. The same effects were obtained in the case of MWCNTs grafted with triethylenetetramine [33] and MWCNTs functionalized with silane. Another approach is to use a combination of MWCNTs with organic and inorganic flame retardant additives to create a synergistic effect [31]. Among inorganic compounds with flame retardant properties, sodium bicarbonate and boric acid could be considered as possible additives to improve thermal stability and reduce flammability of epoxy composites in addition to MWCNTs.

Sodium bicarbonate (NaHCO_3_) is non-toxic and inexpensive material; when heated to a temperature of 60 °C it begins to decompose into sodium carbonate, carbon dioxide, and water:

2NaHCO_3_ → Na_2_CO_3_ + H_2_O + CO_2_ (1)

Upon further heating to 1000 °C, obtained sodium carbonate decomposes into carbon dioxide and sodium oxide:

Na_2_CO_3_→ Na_2_O + CO_2_ (2)

Because of the property of sodium bicarbonate to release CO_2_ gas after decomposition, it is used as the most common chemical blowing agent to produce polymeric foams [34,35], and it can be used as a flame retardant additive in polymers and paints [36].

Boric acid (H_3_BO_3_) is another inorganic compound known as a flame retardant additive for wood, paper, and cotton [37,38]. Boric acid liberates water at the heating above ~100 °C according to the following reactions [39,40]:

2H_3_BO_3_ →2HBO_2_ + 2H_2_O (3)

2HBO_2_ → B_2_O_3_ + H_2_O (4)

The water released during the process of boric acid decomposition reduces polymer temperature of degradation. Boron oxide obtained as a result of reaction (4) forms a glassy film on the burning surface of the polymer, thereby inhibiting diffusion of flammable gases into the flame. Boric acid can provide a greater effect on the improvement of the thermal stability of epoxy composites when it is used in combination with other materials. In our earlier study [41], it was found that, in case of the combination of boric acid (10 wt%) and zeolite (5 wt%), the thermal stability of the epoxy composite was significantly increased, and the maximum decomposition rate at the heating was decreased.

The aim of this work is to study the effects of multi-walled carbon nanotubes, boric acid, and sodium bicarbonate separately, and their combination on the thermooxidative degradation and flammability of the epoxy composites.

## 2. Materials and Methods

The epoxy composites were manufactured by using the epoxy resin DER-331 supplied by Dow Chemical (Stade, Germany), which had the epoxy group content 5200–5500 mmol/kg and average molecular weight of 340 g·mol^−1^. The hardener used was Polyethylenepolyamine (PEPA), supplied by ZAO Uralkhimplast (Nizhny Tagil, Russia). The particles of milled sodium bicarbonate (JSC Bashkir soda company, Sterlitamak, Russia) and boric acid (Tulafarm, Tula, Russia) with a size less of than 64 μm were used as fillers.

The MWCNTs (4060 grade) obtained by Shenzen Nano-Tech Port Co., China, have the diameter of 15–40 nm and the length of 3–5 µm [10].

**Figure 1:**
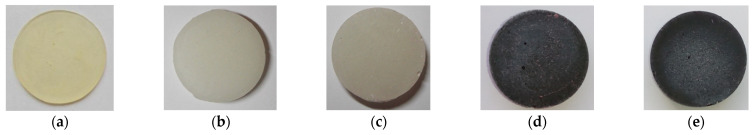
The images of the samples: (**a**) E0; (**b**) E/10B; (**c**) E/10S; (**d**) E/10B/0.5MWCNT; (**e**) E/10S/0.5MWCNT.

The epoxy resin was heated to 55 °C, and then the required amount of the fillers was added into epoxy resin using mechanical stirring for 10 min; afterwards, PEPA was added into mixture. The ratio of epoxy resin and PEPA was 6:1 by mass. The curing of the samples was conducted at room temperature (25 ± 2 °C) for 24 h. The formulations of the studied epoxy composites are given in Table 1. The images of the prepared samples are shown in Figure 1.

The thermal properties of the prepared samples were studied by thermal analyzer STA 449C Jupiter (Netzsch, Germany). The thermogravimetry (TG) and differential scanning calorimetry (DSC) was conducted from 55 to 700 °C at the heating rate of 10 °C min^−1^ in argon–oxygen mixture with an argon flow rate of 180 mL/min and an oxygen flow rate of 20 mL/min. The samples of approximately 5 mg were placed in an Al_2_O_3_ crucible. The studied samples were grinded to powder in mortar for all the experiments.

The Shore D hardness test according to ASTM D2240 at 25 °C was performed on the epoxy composites to measure the hardness. The test was made by placing the studied sample under the indenter of Shore durometer. Then, a load was applied to the sample through the indenter. The tests were repeated five times and mean value is reported.

To evaluate the flammability of the samples, the standard method of experimental determination of the ignition temperature of solid substances and materials was used in accordance with Russian standard GOST 12.1.044-89 “Occupational safety standards system. Fire and explosion hazard of substances and materials. Nomenclature of indices and methods of their determination”. The ignition temperature of the epoxy resin and composites was determined using the installation, which is a vertical electric furnace with two coaxially located cylinders made of quartz glass. The spiral electric heaters with a total power of at least 2 kW are wound on the cylinders, allowing the creation of a working zone temperature of 600 °C during 40 min. The principle of the installation is based on setting the temperature regime in the reaction chamber and the action of burner flame on the samples after introducing them into the reaction chamber; afterwards, the temperature characteristics of the material were monitored. The samples prepared were of cylindrical shape with a diameter of 31.4 mm, and the weight of each sample was 6 ± 0.1 g. At least six samples for each formulation were tested for the good reproducibility.

## 3. Results

### 3.1. TG Results

**Figure 2:**
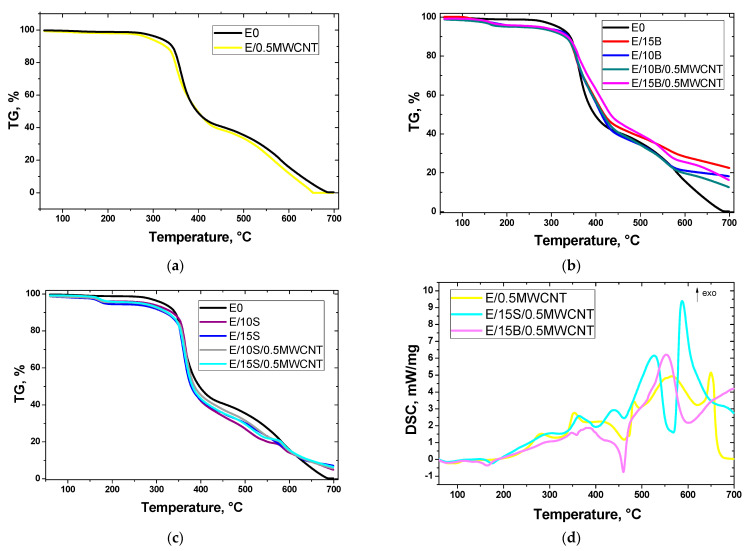

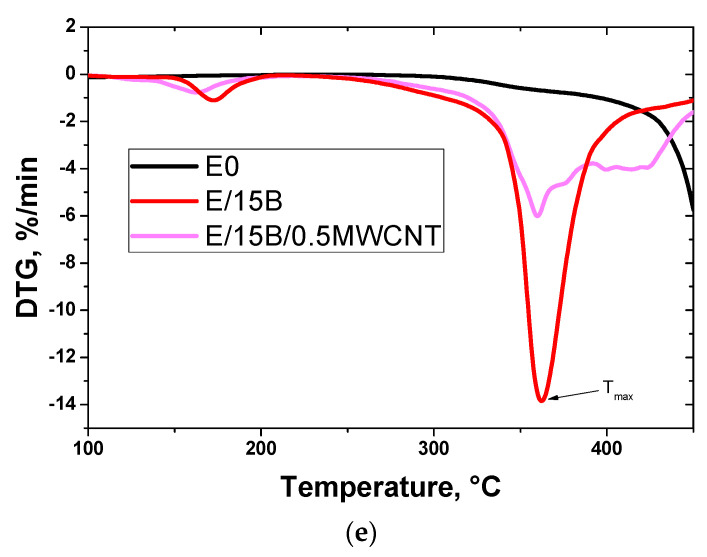
TG curves of epoxy composite samples E0 and E/0.5MWCNT (**a**), with the addition of boric acid and MWCNTs (**b**), sodium bicarbonate and MWCNTs (**c**), DSC curves of the samples E0 and E/0.5MWCNT (**d**) and DTG curves of the samples for *T*_max_ determination (**e**).

The thermal behavior of the epoxy composites filled with MWCNTs, boric acid, sodium bicarbonate, and their combination at the heating was investigated by means of TG analysis. The TG curves of the studied epoxy composites are shown in Figure 2.

The data obtained from the TG curves of the prepared epoxy composites are given in Table 2. The initial temperature of degradation is considered as the temperature at which the mass loss is 5 wt% (*T*_5_). The midpoint temperature *T*_50_ corresponds to a 50% mass loss. The temperature *T*_90_ corresponds to a 90% mass loss. The thermooxidative degradation of the neat epoxy E0 finished nearly at 690 °C, and the residue is 0.2%. Therefore, we considered the residue at 690 °C to evaluate the thermal stability of the composites.

Incorporation of 0.5 wt% MWCNTs into epoxy matrix was not found to lead to the improvement in thermal stability of the sample E/0.5MWCNT. After the addition of MWCNTs to epoxy resin, the temperature *T*_5_ decreased by 23.8 °C. The temperature *T*_50_ slightly increased by 1.4 °C. However, the temperature *T*_90_ for the sample E/0.5MWCNT decreased by 19.1 °C in comparison with that of the sample E0, and is linked with oxidation of filler at these temperatures. The effect of increased mass loss of MWCNT composites at 250–350 °C compared with pure epoxy was also observed in [42]; the authors attribute this effect to the presence of a small amount of uncured epoxy in the epoxy/MWCNT sample.

**Figure 3:**
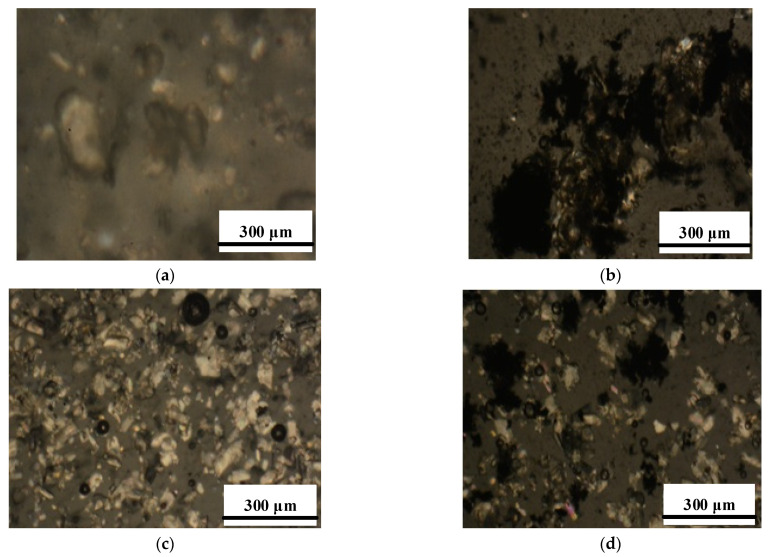
Optical microscopy images of epoxy composites: (**a**) E/15B, (**b**) E/15B/0.5MWCNT, (**c**) E/15S, (**d**) E/15S/0.5MWCNT.

Both samples E0 and E/0.5MWCNT practically completely decomposed at 690 °C. This result can be probably explained by the poor distribution of MWCNTs in the epoxy matrix. Optical micrographs of composites are presented in Figure 3. It was found that MWCNTs form bundles in epoxy filled with boric acid or sodium carbonate.

The initial temperature of degradation *T*_5_ of the boric acid and sodium bicarbonate is much lower than that of the epoxy resin; as a result, the presence of these fillers in the epoxy composite leads to a decrease in the *T*_5_ of the composites. During combustion, these fillers can dilute the flammable gases by carbon dioxide (sodium bicarbonate) and reduce the temperature in the flame zone via water (boric acid and sodium bicarbonate), thereby inhibiting the combustion process. The thermal behavior of epoxy resin ED-20 filled with boric acid at the heating in air and in argon was investigated in [41,43]. The TG curves of the samples E/10B and E/15B (Figure 2b) displayed two steps of degradation accompanied by the release of water in the process of boric acid decomposition upon heating corresponding to chemical reactions (3) and (4): The first step of the process of boric acid decomposition is between 120 and 150 °C with 30% of mass loss; the second step, with 15% of mass loss, is between 150 and 350 °C [43]. The results of TG analysis of the sodium bicarbonate at the heating in air revealed that the decomposition process of sodium bicarbonate is characterized by two endothermic effects with the temperature maxima of 162 and 852 °C that correspond to chemical reactions (1) and (2). The mass loss for the first stage of the sodium bicarbonate decomposition observed in the temperature range from 120 to 189 °C is 39%. Similarly, for samples E10S and E15S, the TG curves in the investigated temperature range exhibit a degradation stage corresponding to the first step of the sodium bicarbonate decomposition upon heating.

The introduction of sodium bicarbonate and boric acid as fillers into epoxy matrix leads to a decrease in the initial temperature of degradation *T*_5_ of the samples via the early decomposition of these substances. When the samples filled with boric acid E/10B and E/15B are heated above ~360 °C, they become more stable compared to the unfilled sample E0. The temperature *T*_50_ of the samples E/10B and E/15B is higher than that of the sample E0 by 15.5 and 21.7 °C, respectively. The residue at 690 °C of the samples E/10B and E/15B is higher in comparison with that of the sample E0 by 18.3 and 22.9%, respectively. The combination of boric acid and MWCNT in the samples E/10B/0.5MWCNT and E/15B/0.5MWCNT resulted in their greater resistance to heating above the temperature 350 °C than the neat epoxy sample E0 and E/0.5MWCNT as well. The temperatures at which the samples E/10B/0.5MWCNT and E/15B/0.5MWCNT lose 50% of the mass upon heating are the highest; are 19 and 35.7 °C higher than that for the unfilled sample E0, respectively; and are 3.5 and 14 °C higher than that for the samples E/10B and E/15B, respectively. However, the residue at 690 °C for the E/10B/0.5MWCNT and E/15B/0.5MWCNT is lower compared to that of samples E/10B and E/15B. The endothermic DSC peak with the maximum around 460 °C is attributed to boric acid decomposition (Figure 2d).

The samples filled with sodium bicarbonate E/10S and E/15S showed a lower thermal stability than the neat epoxy resin E0. The temperature *T*_50_ of the samples E/10S and E/15S is lower than that of the sample E0 by 15.3 and 18.9 °C, respectively. In contrast, the residue at 690 °C of the samples E/10S and E/15S is higher in a comparison with that of the sample E0 by 5.4 and 7.3%, respectively. The temperature at which the samples E/10S and E/15S lose 90% of the mass *T*_90_ is also higher than that for the sample E0. The combination of sodium bicarbonate and MWCNT in the samples E/10S/0.5MWCNT and E/15S/0.5MWCNT resulted in low thermal stability upon the heating in comparison with the samples E0 and E/0.5MWCNT. The *T*_50_ of the samples E/10B/0.5MWCNT and E/15B/0.5MWCNT is lower than that of the sample E0 by 11.2 and 15.7 °C, respectively. Moreover, the residue at 690 °C for the sample E/10S/0.5MWCNT is higher by 0.6 °C compared to that of the samples E/10S, whereas the residue for the sample E/15S/0.5MWCNT is lower by 0.6 °C than that for the sample E/15S.

The value of *T*_max_, the temperature corresponding to the maximum of peak of differential TG curve (DTG) is determined according to Figure 2e and is presented in Table 2. *T*_max_ ranged from 355.0 to 366.9 °C, and the highest value was shown by E/10S sample. As seen from DTG curves, addition of boric acid and MWCNTs makes the DTG peak complex, with a broad temperature range of weight loss. Instead of metallic particles used for enhancement of epoxy resin degradation behavior [44], the addition of MWCNTs and boric acid expands the degradation process during heating.

Glass transition temperature *T*_g_ shown in Table 2 increases when adding MWCNTs in pure epoxy due to improving of cross-linking. Addition of boric acid and sodium bicarbonate induces the increase of *T*_g_ to 160–175 °C (E/10B, E/15B) and 170–175 °C (E/10S, E/15S). Generally, the glass transition temperature is higher for composites containing sodium bicarbonate and MWCNTs (E/10S/0.5MWCNT—175.9 °C, E/15S/0.5MWCNT—172.7 °C) compared to composites containing boric acid.

### 3.2. Hardness

Hardness is defined as the property of material to resist penetration, abrasion, and indentation. The measured Shore D hardness values of the studied samples are given in Table 3.

From these results, the sample E/0.5MWCNT shows the highest hardness among all the samples. The hardness of the sample E/0.5MWCNT is 10.5% higher than that of the neat epoxy E0. Incorporation of MWCNTs into the epoxy matrix increases the cross-linking density of epoxy that restricts the motion of polymer chains and resulted in increased hardness [45,46]. The samples filled with both boric acid and sodium bicarbonate individually and in combination with MWCNTs have the lower hardness compared to the neat epoxy E0 and the sample E/0.5MWCNT. The addition of boric acid or sodium bicarbonate reduces hardness, since filler particles at a sufficiently high loading hinder the cross-linking of epoxy resin chains, as it was reported by various authors [5,47,48]. Probably, the decrease in the hardness can be attributed to the non-uniformity of the fillers distribution in the composites and the void formation during the sample preparation of the composites with the higher fillers content [49,50].

### 3.3. Flammability Test

Table 4 shows the results of the experimental determination of the ignition temperature of the epoxy composites and the average time-to-ignition of the samples.

**Figure 4:**
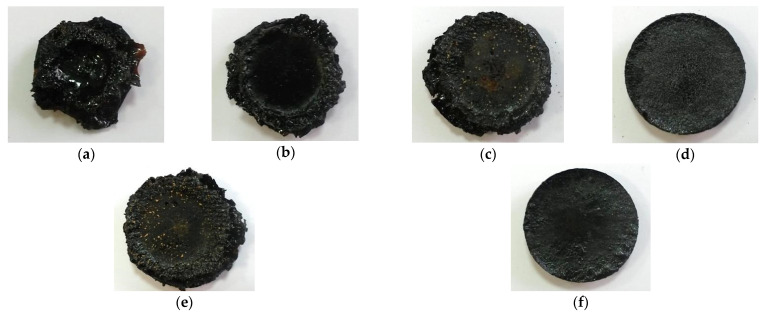
Digital images of the residue chars after flammability test: (**a**) E0; (**b**) E/0.5MWCNT; (**c**) E/15B; (**d**) E/15S; (**e**) E/15B/0.5MWCNT; (**f**) E/15S/0.5MWCNT.

The digital images of the samples after flammability test are given in Figure 4.

The ignition temperature is the minimum temperature at which a combustible substance when heated takes fire in air and continues to burn. The time-to-ignition is defined as the minimum exposure time required for the sample to ignite and sustain flaming combustion. It should be noted that ignition tests data are closer to practical application of epoxy composites under the influence of high temperatures in fire conditions than that of TG analysis data.

The neat epoxy resin E0 melts and flows upon heating and burning, as shown in Figure 3a. The sample E/0.5MWCNT showed the maximum ignition time. These results can be attributed to the active role of MWCNTs filler in the formation of a network-structured protective layer [31]. The introduction of boric acid and sodium bicarbonate individually leads to an increase in the ignition temperature of the samples compared to the unfilled sample E0. The combination of boric acid (15 wt%) with MWCNTs leads to an increase in the ignition temperature of the sample compared to a sample filled with MWCNTs only. The endothermic process of boric acid decomposition with the release of water leads to cooling of the composite surface. Moreover, the formation of a boron oxide glassy phase as can be seen in Figure 3c,e for the samples E/15B and E/15B/0.5MWCNT provides a physical barrier effect. The protective glassy layer of boron oxide diminishes the heat flow into epoxy matrix and prevents further degradation of epoxy [51,52].

The maximum ignition temperature was observed in sample E/10S. The incorporation of 15 wt% of sodium bicarbonate led to a decrease in the ignition temperature by 2 °C. Sodium bicarbonate releases carbon dioxide and water upon burning the composites and causes formation of the dense char residue with a large number of small bubbles, uniformly distributed in the sample volume. This structure is typical for all the samples filled with sodium bicarbonate (Figure 3d,f). The incorporation of 10 wt% boric acid and sodium bicarbonate in combination with MWCNTs did not lead to an improvement in the flammability characteristics. The ignition temperature for the samples E/10B/0.5MWCNT and E/15S/0.5MWCNT is lower compared to the sample filled with only MWCNTs, but higher than that for the unfilled epoxy resin sample. Despite the fact that the sample filled with 10 wt% of sodium bicarbonate has the highest ignition temperature (320 °C), the combination with MWCNTs in the sample E/10S/0.5MWCNT led to a reduction of the ignition temperature. Moreover, the samples filled with sodium bicarbonate separately and in combination with MWCNTs have the lowest time-to-ignition values.

## 4. Conclusions

In this research, the thermal properties (initial temperature of degradation, midpoint temperature, temperature of 90% mass loss, residue at 690 °C) of the epoxy composites filled with MWCNTs (0.5 wt%), boric acid and sodium bicarbonate (10 and 15 wt%) separately, as well as in combination with MWCNTs, were compared to those of the control unfilled sample. The addition of 0.5 wt% MWCNTs in the epoxy matrix did not improve the thermal stability of the epoxy composite. The improvement in the thermal properties can be achieved by the combined use of MWCNTs and boric acid. The thermooxidative degradation of the samples filled with boric acid passes more slowly and more evenly. The ignition temperature and time-to-ignition of the neat epoxy resin and composites was determined. Based on the results of thermal analysis, it was concluded that sodium bicarbonate in the studied concentration ranges (10 and 15 wt%), despite the high ignition temperature value, is not recommended to be used as filler capable of increasing the thermal stability and reducing the flammability of epoxy polymers. In addition, the incorporation of boric acid and sodium bicarbonate to the epoxy matrix resulted in a decrease in Shore D hardness. Thus, the effect of additives deteriorating the mechanical properties of composites must be taken into account when developing recommendations for the practical use of epoxy composites. The obtained composites with enhanced thermal stability can be used as adhesives, coatings, housings, details of some machines which can be subjected to impact of heating. The fillers used in the preparation of epoxy composites make it possible to delay the degradation process upon heating.

## Figures and Tables

**Table 1:** Formulations of epoxy composites (the concentration of fillers are parts per hundred parts of pure resin (phr)).

No.	Sample	Epoxy Resin	H_3_BO_3_	Na_2_CO_3_	MWCNTs
1	E0	100	0	0	0
2	E/0.5MWCNT	100	0	0	0.5
3	E/10B	100	10	0	0
4	E/15B	100	15	0	0
5	E/10S	100	0	10	0
6	E/15S	100	0	15	0
7	E/10B/0.5MWCNT	100	10	0	0.5
8	E/15B/0.5MWCNT	100	15	0	0.5
9	E/10S/0.5MWCNT	100	0	10	0.5
10	E/15S/0.5MWCNT	100	0	15	0.5

**Table 2:** Thermal properties of epoxy composites.

Sample	*T*_5_ (°C)	*T*_50_ (°C)	*T*_90_ (°C)	Residue at 690 °C (%)	*T*_max_ (°C)	*T_g_* (C)
E0	314.9	397.1	627.8	0.2	361.2	126.1
E/0.5MWCNT	291.1	398.5	608.7	0.0	358.3	139.8
E/10B	229.3	412.6	–	18.5	355.0	174.6
E/15B	266.1	418.8	–	23.1	354.7	160.2
E/10S	279.2	381.8	640.3	5.6	366.9	174.5
E/15S	181.9	378.2	636.1	7.5	360.1	170.4
E/10B/0.5MWCNT	222.4	416.1	–	13.4	357.1	169.5
E/15B/0.5MWCNT	272.7	432.8	–	17.4	359.1	163.8
E/10S/0.5MWCNT	260.7	385.9	640.4	6.2	363.2	175.9
E/15S/0.5MWCNT	265.4	381.4	647.1	6.9	362.2	172.7

**Table 3:** Hardness values of samples.

Sample	Shore D
E0	81.2 ± 2.7
E/0.5MWCNT	85.3 ± 2.5
E/10B	79.6 ± 4.3
E/15B	74.7 ± 2.9
E/10S	79.6 ± 4.3
E/15S	75.2 ± 3.4
E/10B/0.5MWCNT	75.0 ± 2.6
E/15B/0.5MWCNT	73.9 ± 3.4
E/10S/0.5MWCNT	75.9 ± 1.8
E/15S/0.5MWCNT	78.4 ± 1.9

**Table 4:** Ignition temperature and time-to-ignition.

Sample	Ignition Temperature, °C	Time-to-Ignition
E0	308	7 min 17 s
E/0.5MWCNT	315	7 min 26 s
E/10B	318	7 min 1 s
E/15B	318	7 min 12 s
E/10S	320	6 min 36 s
E/15S	318	6 min 32 s
E/10B/0.5MWCNT	312	7 min 1 s
E/15B/0.5MWCNT	318	7 min 13 s
E/10S/0.5MWCNT	306	6 min 53 s
E/15S/0.5MWCNT	312	6 min 16 s

## Data Availability

Data is contained within the article.
